# Modern radiation therapy and potential fertility preservation strategies in patients with cervical cancer undergoing chemoradiation

**DOI:** 10.1186/s13014-015-0353-4

**Published:** 2015-02-22

**Authors:** Pirus Ghadjar, Volker Budach, Christhardt Köhler, Andreas Jantke, Simone Marnitz

**Affiliations:** Department of Radiation Oncology, Charité Universitätsmedizin Berlin, Augustenburger Platz 1, 13353 Berlin, Germany; Department of Gynecology, Charité Universitätsmedizin Berlin, Berlin, Germany

**Keywords:** Cervical cancer, Radiation therapy, Fertility preservation, Chemotherapy

## Abstract

Young patients with cervical cancer who undergo chemoradiation might be interested in fertility preservation, not only dependent upon the use of a gestational carrier as maybe achieved by the use of ovarian transposition and cryo-conservation of oocytes or ovarian tissue, but may prefer to carry pregnancy to term after cancer treatment. The latter approach is a non-established concept needing both modern radiation therapy approaches as well as modifications -if at all possible- in current recommendations for target volume delineation to spare dose to the unaffected uterus. Future strategies to serve selected patients in this respect should only be conducted in prospective clinical evaluations and are critically discussed in this article.

## Introduction

Cervical cancer is one of the most common cancers diagnosed in female patients under the age of 40 years [1]. Successful treatment leading to cure is the major concern for most patients. However, for young patients, preservation of fertility and pregnancy related complications after treatment are also of importance. Therefore, if present, the desire to cure the cancer and additionally achieve fertility preservation poses several important considerations both for the patient and the interdisciplinary oncologic team. Due to the trend of delaying childbearing in Western societies the interest in fertility preservation might be rising in female cancer patients. For patients with cervical cancer who have to undergo chemoradiation, preservation of ovarian function and preservation of the functionality of endometrial and myometrial structures are of importance but remain a challenge in clinical practice. Overcoming these problems would offer selected patients the chance for both, cancer control and preservation of fertility, including nidation of the ovule in their own uterus e.g. carrying a child to term. Recent interdisciplinary approaches for fertility preservation in cervical cancer treatment are critically discussed.

## Review

### The preservation of ovarian function, cryo-conservation and ovarian transposition

A successful pregnancy is dependent upon a functional hypothalamic-pituitary-ovarian axis and the ability of the uterus to receive nidation and to accommodate normal growth of the fetus to term [2]. The nonrenewable pool of ovarian primordial follicles declines through atresia with age, from around 2 million at birth to 500.000 at menarche. Further decrease of the number of primordial follicles is associated with an increased difficulty of spontaneous conception during lifetime [3,4]. This natural decrease can be aggravated by chemotherapy as well as radiation therapy causing direct DNA damage to follicles. Ovarian tissue is very sensitive to radiation [5]. It was estimated that ≤ 2 Gy will destroy half of immature oocytes [4,6] and 4 Gy produces infertility in a third of young women and in almost all women over 40 years of age [7]. Childhood Cancer Survivor Study (CCSS) demonstrated that the occurrence of acute ovarian failure was not only associated with older age at diagnosis but also with the conduction of abdominal or pelvic radiation therapy, especially those who received at least 10 Gy to the ovaries [8].

Preservation of ovarian function is an emerging medical, emotional and quality of life issue for pre-menopausal women affected by cervical cancer [9]. However, methods of ovarian preservation are often underused (only in 31 out of 108 patients) as demonstrated by Han et al. in a retrospective, single center study [10]. Ovarian function can be preserved either by cryo-conservation and re-transplantation of ovarian tissue after oncologic treatment or by ovarian transposition (OT).

In current practice a proportion of young cervical cancer patients undergo cryo-conservation of unfertilized oocytes after appropriate ovarian stimulation [11]. Another established option which however requires a partner is in vitro fertilization (IVF) and cryo-preservation of embryos, which is not regulated by legislation in several countries [9]. Alternatively ovarian tissue might be cryo-preserved and later be re-implanted, preferably by an orthotopic approach, a procedure which requires no partner and no hormonal stimulation [12]. Whether ovarian suppression through treatment with gonodotropin-releasing hormone (GnRH) agonists or antagonists during chemotherapy might help to maintain fertility is controversially discussed [13]. First live birth after cryo-preservation of ovarian tissue followed by transplantation was described in 2004 in a woman with Hodgkin’s lymphoma [12]. To the best of our knowledge until today the birth of 18 healthy babies has been reported after transplantation of frozen-thawed human ovarian tissue [14]. This promising fertility preservation strategy has also been described in a couple of young women affected by early cervical cancer [15,16].

**Figure 1:**
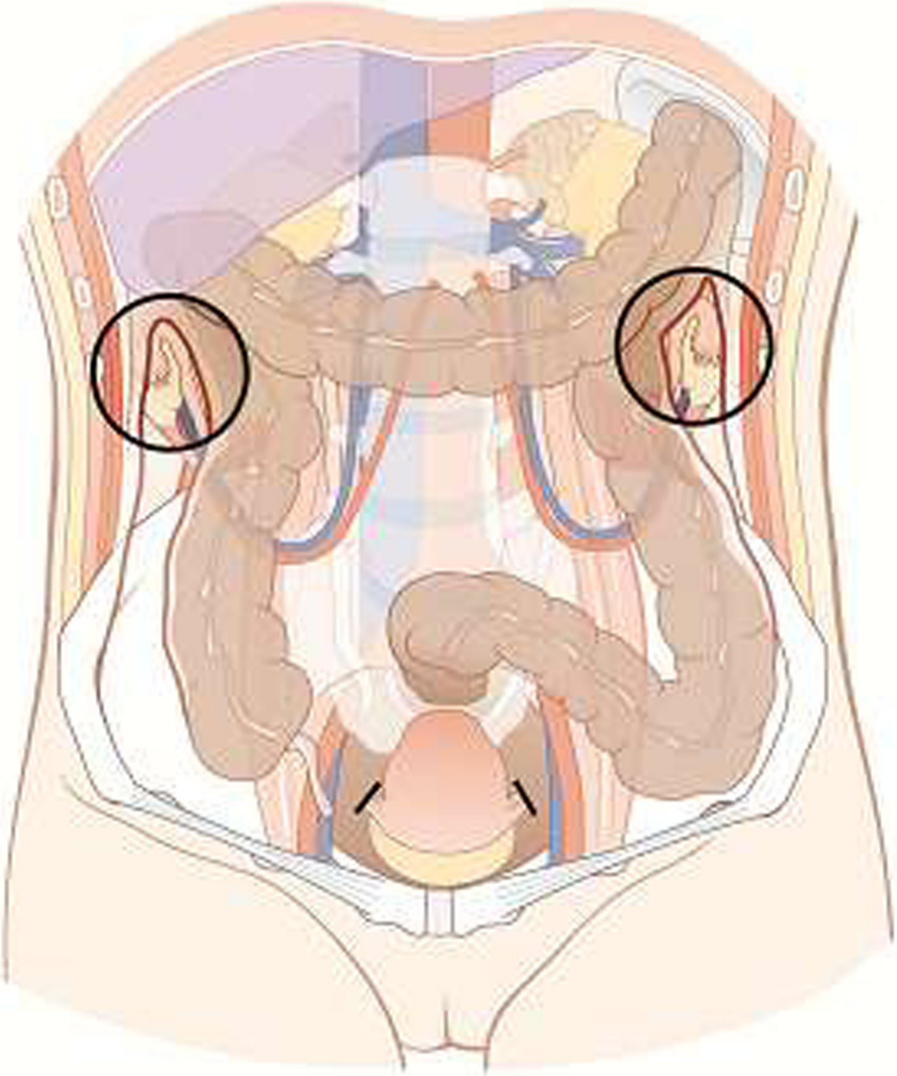
**Transposition of the ovaries with ovarian vessels within the paracolic gutter as high and lateral as possible.**

**Figure 2:**
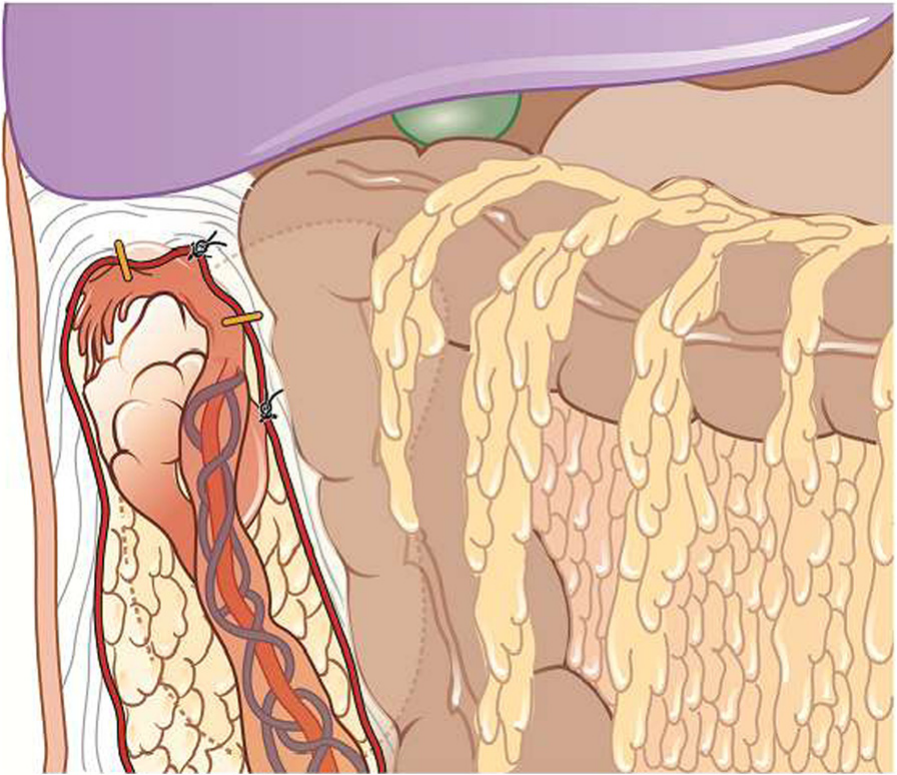
**Fixation of the ovaries with mobilized omentum and identification mark for planning CT using titanium clips (orange).**

In order to reduce the dose applied to the ovaries OT is a surgical procedure to move the ovaries and fallopian tube outside the radiation volume by suturing them within the paracolic gutter as high and lateral as possible (Figures 1 and 2) [17]. Hwang et al. demonstrated that fixation more than 1.5 cm above iliac crest was the most important factor for intact ovarian function [18]. OT can be done during open radical hysterectomy, by laparoscopic approach or more recently used robotic-assisted technique [19,20]. Therefore, maintaining of hormonal function can be achieved in 70%-93% of women younger than 40 years [21-26].

Successful deliveries after IVF stimulated oocyte retrieval from transposed ovary and transfer to surrogate mothers have been described in patient treated for cervical cancer [27-29]. However, metastases in transposed ovaries also may occur occasionally [30-32]. Data for prevalence of ovarian metastases in patients with cervical cancer in the literature vary between 0% and 15%. Known risk factors for ovarian spread are tumor size, histologic type (squamous versus adenocarcinoma), grading, lymphovascular space involvement and haemovascular involvement, all of those having been discussed controversially [24,33-36], however bilateral-oophorectomy is not part of the standard surgical management of cervical cancer. Therefore, benefit of keeping hormonal function must be balanced against (low) risk of ovarian metastases. We believe that OT should be offered to all patients with cervical cancer younger than 40 years without morphologic abnormalities in the ovaries, stages I-IIB of disease with indication for primary or adjuvant chemo-radiation and without risk for familial ovarian cancer after informed consent.

### Chemotherapy related ovarian failure

Another reason of ovarian failure might be the application of chemotherapy in combination with radiation therapy. Most of the available literature on use of chemotherapy and consecutive infertility is limited because of reporting amenorrhea as a surrogate measure of infertility. Generally, a decrease of the total number of primordial follicles could be detected after application of chemotherapeutic drugs and it appears that alkylating agents have the highest risk of permanent amenorrhea, while the risk after cisplatin-containing chemotherapy which is the drug of choice in the treatment of cervical cancer, is considered to be of intermediate risk for infertility [2,13]. Furthermore it has been described that multi-agent chemotherapy without radiation therapy was not associated with the occurrence and outcome of pregnancies [37].

### Modern ovarian and uterine sparing techniques in radiation oncology

Current pre-chemoradiation fertility preserving strategies such as cryo-conservation of oocytes or ovarian tissue and limitation of the dose applied to the ovaries [3], ultimately were depending on the use a surrogate mother, as uterine dysfunction after pelvic radiation therapy was assumed to preclude to carry a pregnancy to term.

However, due to the availability of newer radiation therapy techniques including intensity modulated radiation therapy (IMRT) as well as CT and MRT based application of cervical HDR-Brachytherapy or even HDR-Brachytherapy emulating strategies e.g. using robotic radiosurgery, along with improved fertility preservation methods by reproductive medicine experts, today, the question arises whether fertility can be preserved in young patients with cervical cancer including the ability to carry a pregnancy to term. This would have also forensic implications as third-party reproduction using a gestational carrier is illegal in several European countries.

The radiosensitivity of the uterus appears to decrease with advanced age as mentioned above but less data is available from the literature regarding acute and late radiation dose effects on the adult uterus. Milgrom et al. [38] recently described the acute uterine effects after pelvic radiation therapy with a median dose of 50.2 Gy (D95 of the uterus was 30 Gy) in 10 female (7 of which were pre-menopausal) rectal cancer patients who underwent dynamic contrast-enhanced MRI before and 4-7 weeks after radiation therapy. It was found that the median cervical length was reduced after radiation therapy. Interestingly 3 of the analyzed patients who were initially pre-menopausal underwent ovarian transposition and maintained ovarian function after radiation therapy and three other patients were post-menopausal before radiation therapy. Thus in these 6 patients radiation induced ovarian failure would not account for the changes in uterine anatomy. Moreover, in pre-menopausal patients the volume transfer constant (Ktrans) and the extracellular extravascular volume fraction (Ve) were significantly decreased after radiation therapy, suggesting reduced perfusion of the pre-menopausal myometrium after radiation therapy [38].

These functional changes of the uterus could both lead to an impaired implantation of an embryo as well as pregnancy-related complications [3]. The degree of damage has been shown to be dependent on the total radiation dose and it was shown that the pre-pubertal uterus is more vulnerable than the adult uterus to the effect of pelvic radiation therapy, with doses of 14-30 Gy causing uterine dysfunction [3,39,40]. It has been reported after total body irradiation using 8.5-11.7 Gy total dose [41] or 14.4 Gy total dose [2,40] in young female patients, that uterine growth and blood flow were impaired. Likewise, after whole-abdominal radiation therapy using 20-30 Gy during childhood the uterine length was shorter and endometrial thickness was not increased after hormone replacement suggesting irreversible damage to the uterus [39]. Others have described in a cohort of 340 female cancer survivors that after abdomino-pelvic radiation therapy the likelihood to have low-birth-weight infants, premature low-birth-weight infants and the perinatal infant mortality was increased as compared to patients without radiation therapy. These associations were dose dependent and the likelihood to have low-birth-weight infants and perinatal infant mortality were higher in patients receiving >25 Gy as compared to total doses below 25 Gy [42]. Green et al. evaluated the risk of fetal loss among 1915 female cancer survivors of the CCSS. There was a trend for increased miscarriages among women whose ovaries were near or within the radiation volumes compared to patients without radiation therapy. There was also a higher likelihood of low-birth-weight infants found in patients who were treated with pelvic radiation therapy [37]. Signorello et al. [43] analyzed the risk of preterm birth among 1264 female cancer survivors of the CCSS and found an increasing risk of preterm birth with increasing cumulative dose to the uterus. In contrast to the children of survivors who did not receive any radiation therapy (among whom 19.6% were born preterm), preterm birth was reported for 26.1% of the children of survivors who received uterine doses in the range of 0.5-2.5 Gy (odds ratio (OR) = 1.8, 95% confidence interval (CI) = 1.1 to 3.0; *P* = .03), for 39.6% of the children of survivors who received uterine doses in the range of 2.5-5 Gy (OR = 2.3, 95% CI = 1.0 to 5.1; *P* = .04), and for 50.0% of the children of survivors who received uterine doses higher than 5 Gy (OR = 3.5, 95% CI = 1.5 to 8.0; *P* = .003). After stratification according to whether the treatment occurred pre- or post-menarche it was found that the association between uterine dose and preterm birth appeared to be stronger for survivors exposed before menarche (for >2.5 Gy, OR = 4.9, 95% CI = 1.7 to 13.9; *P* = .003) than those exposed after menarche (for >2.5 Gy, OR = 1.9, 95% CI = 0.7 to 4.9; *P* = .21) suggesting that the uterus is less sensitive to radiation dose with higher age. Additionally, increasing dose to the uterus was found to be related to the risk of low-birth weight (no radiation therapy 7.6%; uterine dose 2.5-5 Gy 25.5%; uterine dose >5 Gy 36.2% low-birth weight infants, respectively).

**Figure 3:**
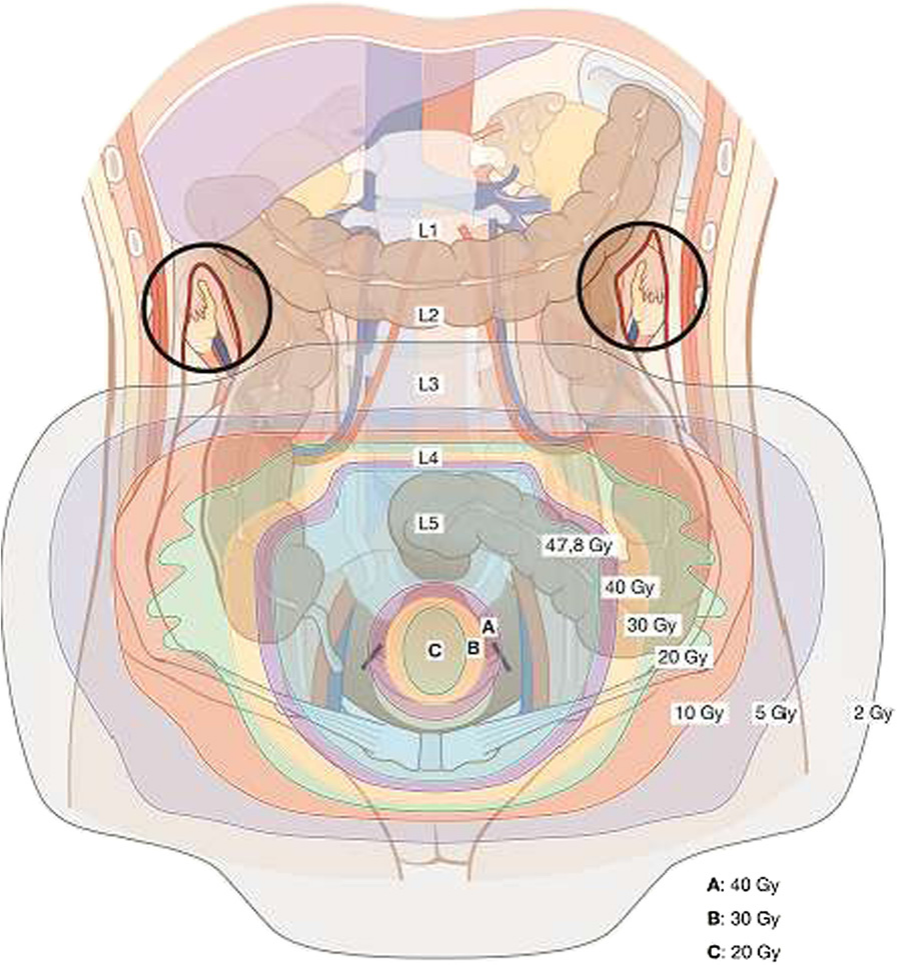
**Isodoses of the prescribed dose (47.8 Gy) in the target volume decreasing to the periphery and to the ovaries (in black circles) to < 2 Gy between second (L2) and third (L3) lumbar vertebrar.** Selective dose reduction within the intact uterus from 40 Gy **(A)** to 30 Gy **(B)** in the periphery to 20 Gy **(C)** in the inner layer of the myometrium and endometrium.

Sophisticated external beam irradiation techniques (IMRT, volumetric arc therapy and helical tomotherapy) offering by means of “dose painting” and sharp dose gradients against normal tissue a considerable dose reduction not only to the transposed ovaries but also to the uterus itself. Figure 3 illustrates the isodoses in a patient undergoing concurrent chemoradiation with RapidArc® technique. The 95% isodose (95% of 50.4 Gy = 47.8 Gy) covers the target volume including the cervix and the pelvic lymph nodes. A selective dose reduction can be achieved for the inner myo- and endometrial structures to avoid myometrial shrinkage and endometrial atrophy after radiation therapy. A clear dose correlation for endometrial functionality had not been established yet. According to glandular function of other organs (e.g. parotid gland) we try to keep the mean dose <20-25 Gy (Figure 3 A, B and C).

The current recommendations for target volume delineation using IMRT in cervical cancer however recommend to include the entire uterus into the clinical target volume (CTV) because uterus and cervix are embryologically one unit with interconnected lymphatics and no clear separating fascial plane. It was not clear how often and where intrauterine recurrences occur after chemoradiation for cervical cancer [44]. However, the question whether the uterus has to be included completely or partially into the CTV was discussed controversially amongst involved experts. 42 percent of survey respondents felt that it was not always necessary to include the entire uterus in the CTV and it was stated that excluding a portion of the corpus would be an option for selected cases when sufficient data are available regarding the incidence and exact location of uterine recurrence after conservative surgical procedures [44]. On the other hand, the assumption that all structures of one embryologic compartment should be included into the target remains controversial since radical trachelectomy has been demonstrated excellent results while preserving the uterus and oophorectomy is not considered a standard procedure in this setting [45]. However, the results of trachelectomy might not be transferred to patients undergoing chemoradiation as the latter usually have more advanced disease.

When IMRT should be used to spare healthy uterine tissue, an appropriate management of uterine motion is crucial, as interfractional uterine movement has been well described by others [46]. Besides bladder and rectum filling recommendations we recommend daily soft-tissue imaging with correction for interfractional motion or adaptive replanning if deemed necessary.

With the use of MRI guided brachytherapy, the extent of the macroscopic tumor can be exactly determined and the uninvolved corpus uteri should not be part of the target volume [47].

Furthermore we have shown that HDR-Brachytherapy (which is regarded as the standard technique for dose escalation to the cervix) can be emulated by Cyberknife robotic radiosurgery with an excellent target coverage and steep dose gradients toward normal surrounding tissues [48,49]. This approach might further contribute to spare the dose to the uterus. As a cautionary note, it is not definitively known yet to what extent the function of the cervix itself might be compromised, after the package of external beam radiation therapy, HDR-Brachytherapy (or Brachytherapy emulating stereotactic external beam radiation therapy) with a nominal total dose of around 90 Gy, potentially leading to pregnancy related complications.

However, as uterine sparring radiation therapy of cervical cancer is a non-established approach we must be fully aware about the potential disadvantages which might be associated with its use (compromised cancer control maybe even without successful fertility preservation) and treat, if at all, patients within prospective protocols after careful selection. In our center, we offer uterus sparing treatment on request after informed consent to selected patients within a prospective observational trial. We include young women who request fertility preservation and underwent OT with Stage IA2-IB1 disease in the presence of one or more risk factors demanding chemoradiation such as pN1, pM1 (LYM), V1, L1 or G3.

To emphasize it again, patients must be aware about a potentially higher risk of recurrence and the risk for pregnancy related complications before they chose this kind of approach.

## Conclusions

High-precision modern radiation therapy techniques may allow uterine sparing chemoradiation e.g. to reduce the planned dose to the non-affected uterus to below 20-25 Gy. Whether this may preserve fertility, including the ability to carry a pregnancy to term after cancer treatment without compromised cancer control is fully unclear and great caution must remain. It is therefore mandatory, if considered to use this strategy for selected patients, to treat patients within prospective trials.

### Search strategy and selection criteria

References for this Review were identified through searches of PubMed with the search terms “cervical cancer”, “radiation therapy”, “fertility”, and “preservation” from 1990 until February, 2014. Articles were also identified through searches of references of these articles. Only papers published in English were reviewed. The final reference list was generated on the basis of originality and relevance to the broad scope of this review article.

## Abbreviations

CCSS: Childhood cancer survivor study
OT: Ovarian transposition
GnRH: Gonodotropin-releasing hormone
IVF: In vitro fertilization
IMRT: Intensity modulated radiation therapy
Ktrans: Volume transfer constant
Ve: Extracellular extravascular volume fraction
OR: Odds ratio
CI: Confidence interval
CTV: Clinical target volume

## References

[1] Siegel R, Naishadham D, Jemal A (2013). Cancer statistics, 2013. CA Cancer J Clin.

[2] Critchley HO, Wallace WH. Impact of cancer treatment on uterine function. J Natl Cancer Inst Monogr 2005:64–68. 10.1093/jncimonographs/lgi022 15784827

[3] Wo JY, Viswanathan AN (2009). Impact of radiotherapy on fertility, pregnancy, and neonatal outcomes in female cancer patients. Int J Radiat Oncol Biol Phys.

[4] Faddy MJ, Gosden RG, Gougeon A, Richardson SJ, Nelson JF (1992). Accelerated disappearance of ovarian follicles in mid-life: implications for forecasting menopause. Hum Reprod.

[5] Gross E, Champetier C, Pointreau Y, Zaccariotto A, Dubergé T, Guerder C (2010). Normal tissue tolerance to external beam radiation therapy: ovaries. Cancer Radiother.

[6] Wallace WH, Thomson AB, Kelsey TW (2003). The radiosensitivity of the human oocyte. Hum Reprod.

[7] Oncology RotJCfC (1998). Management of Gonadal Toxicity Resulting from the Treatment of Adult Cancer.

[8] Chemaitilly W, Mertens AC, Mitby P, Whitton J, Stovall M, Yasui Y (2006). Acute ovarian failure in the childhood cancer survivor study. J Clin Endocrinol Metab.

[9] Oktay K (2005). Further evidence on the safety and success of ovarian stimulation with letrozole and tamoxifen in breast cancer patients undergoing in vitro fertilization to cryopreserve their embryos for fertility preservation. J Clin Oncol.

[10] Han SS, Kim YH, Lee SH, Kim GJ, Kim HJ, Kim JW (2011). Underuse of ovarian transposition in reproductive-aged cancer patients treated by primary or adjuvant pelvic irradiation. J Obstet Gynaecol Res.

[11] Anderson RAWW (2011). Fertility preservation in girls and young females. Clin Endocrinol (Oxf).

[12] Donnez J, Dolmans MM, Demylle D, Jadoul P, Pirard C, Squifflet J (2004). Livebirth after orthotopic transplantation of cryopreserved ovarian tissue. Lancet.

[13] Lee SJ, Schover LR, Partridge AH, Patrizio P, Wallace WH, Hagerty K (2006). American Society of Clinical Oncology recommendations on fertility preservation in cancer patients. J Clin Oncol.

[14] Dittrich R, Lotz L, Keck G, Hoffmann I, Mueller A, Beckmann MW (2012). Live birth after ovarian tissue autotransplantation following overnight transportation before cryopreservation. Fertil Steril.

[15] Kim SS, Hwang IT, Lee HC (2004). Heterotopic autotransplantation of cryobanked human ovarian tissue as a strategy to restore ovarian function. Fertil Steril.

[16] Kim SS (2012). Assessment of long term endocrine function after transplantation of frozen-thawed human ovarian tissue to the heterotopic site: 10 year longitudinal follow-up study. J Assist Reprod Genet.

[17] Clough KB, Goffinet F, Labib A, Renolleau C, Campana F, de la Rochefordiere A (1996). Laparoscopic unilateral ovarian transposition prior to irradiation: prospective study of 20 cases. Cancer.

[18] Hwang JH, Yoo HJ, Park SH, Lim MC, Seo SS, Kang S (2012). Association between the location of transposed ovary and ovarian function in patients with uterine cervical cancer treated with (postoperative or primary) pelvic radiotherapy. Fertil Steril.

[19] Morice P, Juncker L, Rey A, El-Hassan J, Haie-Meder C, Castaigne D (2000). Ovarian transposition for patients with cervical carcinoma treated by radiosurgical combination. Fertil Steril.

[20] Iavazzo C, Darlas FM, Gkegkes ID (2013). The role of robotics in ovarian transposition. Acta Inform Med.

[21] Gallocher O, Thomas L, Stockle E, Bussières E, Floquet A, Avril A (2002). First surgery followed by vaginal curietherapy in small-volume uterine cervix cancer: an alternative to the association of uterovaginalcurietherapy and surgery. Cancer Radiother.

[22] Barahmeh S, Al Masri M, Badran O, Masarweh M, El-Ghanem M, Jaradat I (2013). Ovarian transposition before pelvic irradiation: indications and functional outcome. J Obstet Gynaecol Res.

[23] Olejek A, Wala D, Chimiczewski P, Rzempoluch J (2001). Hormonal activity of transposed ovaries in young women treated for cervical cancer. Gynecol Endocrinol.

[24] Huang KG, Lee CL, Tsai CS, Han CM, Hwang LL (2007). A new approach for laparoscopic ovarian transposition before pelvic irradiation. Gynecol Oncol.

[25] Ishii K, Aoki Y, Takakuwa K, Tanaka K (2001). Ovarian function after radical hysterectomy with ovarian preservation for cervical cancer. J Reprod Med.

[26] Pahisa J, Martinez-Roman S, Martinez-Zamora MA, Torné A, Caparrós X, Sanjuán A (2008). Laparoscopic ovarian transposition in patients with early cervical cancer. Int J Gynecol Cancer.

[27] Giacalone PL, Laffargue F, Benos P, Dechaud H, Hédon B (2001). Successful in vitro fertilization-surrogate pregnancy in a patient with ovarian transposition who had undergone chemotherapy and pelvic irradiation. Fertil Steril.

[28] Zinger M, Liu JH, Husseinzadeh N, Thomas MA (2004). Successful surrogate pregnancy after ovarian transposition, pelvic irradiation and hysterectomy. J Reprod Med.

[29] Agorastos T, Zafrakas M, Mastrominas M (2009). Long-term follow-up after cervical cancer treatment and subsequent successful surrogate pregnancy. Reprod Biomed Online.

[30] Delotte J, Ferron G, Kuei TL, Mery E, Gladieff L, Querleu D (2009). Laparoscopic management of an isolated ovarian metastasis on a transposed ovary in a patient treated for stage IB1 adenocarcinoma of the cervix. J Minim Invasive Gynecol.

[31] Shigematsu T, Ohishi Y, Fujita T, Higashihara J, Irie T, Hayashi T (2000). Metastatic carcinoma in a transposed ovary after radical hysterectomy for a stage 1B cervical adenosquamous cell carcinoma. Case report. Eur J Gynaecol Oncol.

[32] Morice P, Haie-Meder C, Pautier P, Lhomme C, Castaigne D (2001). Ovarian metastasis on transposed ovary in patients treated for squamous cell carcinoma of the uterine cervix: report of two cases and surgical implications. Gynecol Oncol.

[33] Zhao C, Wang JL, Wang SJ, Zhao LJ, Wei LH (2013). Analysis of the risk factors for the recurrence of cervical cancer following ovarian transposition. Eur J Gynaecol Oncol.

[34] Nakanishi T, Wakai K, Ishikawa H, Nawa A, Suzuki Y, Nakamura S (2001). A comparison of ovarian metastasis between squamous cell carcinoma and adenocarcinoma of the uterine cervix. Gynecol Oncol.

[35] Shimada M, Kigawa J, Nishimura R, Yamaguchi S, Kuzuya K, Nakanishi T (2006). Ovarian metastasis in carcinoma of the uterine cervix. Gynecol Oncol.

[36] Yamamoto R, Okamoto K, Yukiharu T, Kaneuchi M, Negishi H, Sakuragi N (2001). A study of risk factors for ovarian metastases in stage Ib-IIIb cervical carcinoma and analysis of ovarian function after a transposition. Gynecol Oncol.

[37] Green DM, Whitton JA, Stovall M, Mertens AC, Donaldson SS, Ruymann FB (2002). Pregnancy outcome of female survivors of childhood cancer: a report from the Childhood Cancer Survivor Study. Am J Obstet Gynecol.

[38] Milgrom SA, Vargas HA, Sala E, Kelvin JF, Hricak H, Goodman KA (2013). Acute effects of pelvic irradiation on the adult uterus revealed by dynamic contrast-enhanced MRI. Br J Radiol.

[39] Critchley HO, Wallace WH, Shalet SM, Mamtora H, Higginson J, Anderson DC (1992). Abdominal irradiation in childhood; the potential for pregnancy. Br J Obstet Gynaecol.

[40] Bath LE, Critchley HO, Chambers SE, Anderson RA, Kelnar CJ, Wallace WH (1999). Ovarian and uterine characteristics after total body irradiation in childhood and adolescence: response to sex steroid replacement. Br J Obstet Gynaecol.

[41] Holm K, Nysom K, Brocks V, Hertz H, Jacobsen N, Müller J (1999). Ultrasound B-mode changes in the uterus and ovaries and Doppler changes in the uterus after total body irradiation and allogeneic bone marrow transplantation in childhood. Bone Marrow Transplant.

[42] Chiarelli AM, Marrett LD, Darlington GA (2000). Pregnancy outcomes in females after treatment for childhood cancer. Epidemiology.

[43] Signorello LB, Cohen SS, Bosetti C, Stovall M, Kasper CE, Weathers RE (2006). Female survivors of childhood cancer: preterm birth and low birth weight among their children. J Natl Cancer Inst.

[44] Lim K, Small W, Portelance L, Creutzberg C, Jürgenliemk-Schulz IM, Mundt A (2011). Consensus guidelines for delineation of clinical target volume for intensity-modulated pelvic radiotherapy for the definitive treatment of cervix cancer. Int J Radiat Oncol Biol Phys.

[45] Marchiole P, Benchaib M, Buenerd A, Lazlo E, Dargent D, Mathevet P (2007). Oncological safety of laparoscopic-assisted vaginal radical trachelectomy (LARVT or Dargent’s operation): a comparative study with laparoscopic-assisted vaginal radical hysterectomy (LARVH). Gynecol Oncol.

[46] Chan P, Dinniwell R, Haider MA, Cho YB, Jaffray D, Lockwood G (2008). Inter- and intrafractional tumor and organ movement in patients with cervical cancer undergoing radiotherapy: a cinematic-MRI point-of-interest study. Int J Radiat Oncol Biol Phys.

[47] Georg DKC, Hillbrand M, Dimopoulos J, Dimopoulos J, Pötter R (2008). Image-guided radiotherapy for cervix cancer: high-tech external beam therapy versus high-tech brachytherapy. Int J Radiat Oncol Biol Phys.

[48] Marnitz S, Kohler C, Budach V, Neumann O, Kluge A, Wlodarczyk W (2013). Robotic radiosurgery: emulating brachytherapy in patients with locally advanced cervical carcinoma. Radiat Oncol.

[49] Neumann O, Kluge A, Lyubina O, Wlodarczyk W, Jahn U, Kohler C (2014). Robotic radiosurgery as an alternative to brachytherapy for cervical cancer patients. Strahlenther Onkol.

